# Analysis of interprofessional education perceptions at the team level: A study across three student cohorts

**DOI:** 10.1016/j.xjep.2025.100741

**Published:** 2025-03-11

**Authors:** Tina Gunaldo, Kelsey Witmeier, Harun Mazumder, Sharon Duffy, Colette Baudoin, Henry Sauviac, Susanne Straif-Bourgeois, Scott Edwards

**Affiliations:** a Louisiana State University Health Sciences Center New Orleans, USA

**Keywords:** Interprofessional education, Team analysis, Longitudinal, Team-level attitudes

## Abstract

The interprofessional education (IPE) literature showcases many studies reporting results at the all-student and academic program levels. However, if the purpose of IPE is to develop collaborative-practice ready professionals, analysis at the team level would better support the advancement of the IPE literature and future health care goals. Pre-licensure students engaged in a two-year IPE curriculum and individually completed the Student Perceptions of Interprofessional Clinical Education-Revised survey, version 2 (SPICE-R2), at the end of the first and second year. Teams were comprised of health profession students from 12 academic programs associated with the university. Exploratory and longitudinal analyses of the change in mean SPICE-R2 scores from post-year 1 and post-year 2 were conducted. Overall positive changes were seen at the all-student level. However, exploration at a team level revealed more divergent outcomes. Further research into the determinants of these team-level disparities is required to improve IPE curriculum design.

Interprofessional education (IPE) aims to prepare collaborative practice-ready health professionals.^1^ The fast-paced nature of the U.S. healthcare system, which serves increasingly complex patients with multiple comorbidities, requires widespread adoption of interprofessional collaborative practice to create more resilient health systems and promote positive patient outcomes. Graduates trained on interprofessional teams can support improved health service delivery and associated outcomes.^1^ Therefore, developing relevant and impactful IPE evidence is imperative to bolstering support and momentum for cultivating collaborative practice-ready professionals. If IPE has prepared students to effectively work on teams, the educational impacts of IPE programs should be reflected by improved team-level outcomes.

In the United States (U.S.), the Health Professions Accreditors Collaborative (HPAC) advocates for an IPE-integrated curricular approach throughout health-related degree programs.^2^ Since the 2019 HPAC publication, the IPE literature on longitudinal programs and outcomes has grown (Davila et al., 2021;^3^; Miselis et al., 2022;^4^). Current studies evaluate outcomes at an all-student or academic program level but seldom at a team-level. Based on the purpose of IPE and the structure of IPE programs, educational researchers should consider shifting an additional focus to team-level outcomes.

## 1. Background

Team-level perception or attitudinal IPE research is scarce. After searching the Cumulated Index to Nursing and Allied Health Literature (CINAHL) and PubMed databases, only two articles were found.^5,6^ Similarities between these studies included online and in-person IPE learning, a simulation experience focused on a single topic (cancer or cardiac arrest), team size ranging from five to eight persons, learners who were in the latter stages of their curriculum, real-time pre-/post-test design, and the use of validated survey instruments.^5,6^ Ganotice et al.^6^ used the Interprofessional Attitude Scale to study the impact of team attitudes on IPE perceptions. Findings highlighted that team-level attitudes in the Teamwork, Roles, and Responsibilities subscale predicted team performance.^6^ Brown et al.^5^ used the TeamSTEPPS Teamwork Attitude Questionnaire survey to measure the optimal time to deliver cardiac arrest training. The authors found no differences in improving teamwork attitudes when conducting a one-day or a multi-day event.^5^ Both studies have enhanced the IPE literature. However, a significant gap still exists in team-level IPE research, particularly in longitudinal curricular designs.

An initial all-student and team-level exploratory study with one student cohort was conducted and published by a smaller author team from the same southeastern U.S.-based academic health sciences center.^7^ IPE perception findings in this study indicated an overall benefit in engaging in the two-year curriculum at the all-student level.^7^ Total score perception changes significantly improved after engagement in one year and two years of the curriculum.^7^ At the team level, median team score changes from post-year 1 (PY1) and post-year 2 (PY2) either increased, decreased, or demonstrated no change.^7^ Further statistical analysis indicated that changes in mean scores across teams were similar (PY1 and PY2). The differences in team-level scores between PY1 and PY2 were not statistically significant despite individual team IPE perception changes. Findings from a single cohort yielded interest in further investigation of team-level findings across cohorts before, during, and after the COVID-19 pandemic to identify if similar trends persisted. This current study analyzed IPE perceptions change PY1 and PY2 across three cohorts that began the IPE curriculum in 2019, 2020, and 2021.

During the COVID-19 pandemic, many health-related programs moved entirely online. This catalyzed questions among researchers regarding the impact of different education delivery formats on IPE outcomes. In a meta-synthesis review of studies (1971–2015) before the COVID-19 pandemic, researchers compared IPE outcomes from various distance education methods.^8^ There was considerable variation in the types of teaching models using distance education, and other influential variables, such as the length of experience, assessment instrument, number of programs, etc..^8^ The authors concluded more research is needed investigating IPE outcomes related to specific models of delivery, such as online modules, virtual town halls, virtual reality, and asynchronous video case studies.^8^ Published studies since the beginning of the COVID-19 pandemic have compared online to in-person IPE learning outcomes. Findings from these studies have indicated similar outcomes between the two modes of delivery at the all-student and academic program levels,^9–11^ but the team-level outcomes were not evaluated.

## 2. Current study

The current study evaluated all-student and team-level perceptions of three student cohorts after engaging in a two-year interprofessional curriculum. Two research study questions included:

1. How does a two-year curriculum impact IPE perceptions at the all-student level across three student cohorts?
2. How does a two-year curriculum impact IPE perceptions at the team level within each student cohort?

## 3. Methods

The Interprofessional Education Office (IPE) educates students using the two-year curriculum, TEAM UP COMPASSION, COMMUNICATION, COLLABORATION^®^ (TEAM UP^™^). TEAM UP^™^ activities and curriculum are grounded in the Interprofessional Education Collaborative competencies,^12^ the meta-model for interprofessional development,^13^ the interprofessional socialization framework,^14^ and the 7 C’s of team science (Salas, 2016). To support the advancement of interprofessional science (Xyrichis, 2020) through replication and generalizability of studies, information describing the curriculum is outlined in Table 1, as recommended by Gunaldo et al..^15^

Three student cohorts in this study included 1) Cohort 2019, 2) Cohort 2020, and 3) Cohort 2021. The year of the cohort indicates the year when the student cohort began the IPE curriculum. Table 2 provides the semester engagement of each cohort. The three student cohorts engaged in IPE synchronously: 1) on-campus and/or 2) via a video web-based platform. Student team engagement was on campus during the 2019–2020 academic year. As the COVID-19 pandemic catalyzed and sustained, the curriculum was delivered through a web-based video platform for the 2020–2021 and 2021–2022 academic years. Student team engagement reverted to on-campus in the 2022–23 academic year. Table 2 provides an overview of the format by student cohort and academic year semester.

post.

As a curriculum component, students were required to complete the Student Perceptions of Interprofessional Clinical Education-Revised survey, version 2 (SPICE-R2). The SPICE-R2 was placed in the institution’s web-based learning management platform, and students submitted their survey after the last spring semester session of the academic year was completed. The LSU Health Sciences Center New Orleans’ Institutional Review Board (IRB) approved this research (IRB #10106).

### 3.1. Participants

Health professional students in this study were from 12 undergraduate and graduate degree programs at a southeast U.S. academic health sciences center. Graduate programs included audiology, dentistry, medicine, occupational therapy, physical therapy, physician assistant, public health, and speech-language pathology. Undergraduate programs included cardiovascular sonography, dental hygiene, medical laboratory science, nursing, and respiratory therapy. Sixty teams were created through random assignment, prioritizing program diversity. Team size ranged from 10 to 15 students, and students remained on the same team for two years.

Students included in the study participated in TEAM UP^™^ for two academic years and completed the SPICE-R2 questionnaire at the end of each academic year (both PY1 and PY2 of the curriculum). Cohort 2019 included 675 students, Cohort 2020 included 633 students, and Cohort 2021 included 601 students; only one student was excluded from the study.

### 3.2. Survey instrument

At the end of each academic year, students completed the SPICE-R2 using a retrospective pre-/post-test design. The SPICE-R2 is a reliable instrument (α = 0.85), including ten statements to which students indicate their agreement using a 5-point Likert scale.^16^ Three subscales were evaluated in the survey, including teamwork (TW), patient outcomes (PO), and roles/responsibilities (RR).^16^

### 3.3. Analysis

We used a retrospective pre-/post-test design to minimize response shift bias noted in real-time pre-/post-test design surveys.^17^ We calculated SPICE-R2 scores (Total Score [TS], TW, PO, RR) for each student cohort by subtracting the retro-pre from the post score at the end of Year 1 and Year 2 of the curriculum.

Team-level (PY1 and PY2) scores were first explored using boxplots to visualize and compare the distributions based on the data’s center and variability between each year within the same student cohort and across cohorts. The boxplots were summarized by calculating the percentage of teams rendering changes in median and interquartile range (IQR) and presented in bar graphs (Supplement). An increase in the median for the same team from PY1 to PY2 implied a team improvement in IPE perceptions. Teams with members with similar changes in IPE perceptions would have a decreased IQR when comparing PY1 to PY2 box plots. However, an increase in IQR implied team members had more diverse changes in IPE perceptions at PY2 than in PY1.

Next, the statistical significance of the findings from exploratory analyses was measured using repeated-measure analysis of variance (RM-ANOVA) to account for within-student differences across two time points (PY1 and PY2) within each cohort. Specifically, the impact of a two-year curriculum on IPE perceptions (TS, TW, RR, and PO) at the all-student level across three cohorts was investigated using a two-way RMANOVA. We interpreted the interaction of the explanatory variables time (PY1 and PY2) and cohort (Cohort 2019, 2020, and 2021) to assess if the effect of time on students’ perceptions significantly varied across the three cohorts. The normality and sphericity assumptions of repeated-measure ANOVA were investigated. Mauchly’s Test checked the sphericity assumption, and Greenhouse–Geisser correction was applied to the F-statistic if a lack of sphericity was observed.

To investigate the impact of the two-year curriculum on IPE perceptions at the team level across three cohorts, we performed a two-way RM-ANOVA within each cohort and interpreted the interaction effects of time (PY1 and PY2) and team (team 1 to team 60). Education delivery format was not included as a separate variable because of its strong correlation with cohort and the risk of multicollinearity. However, the cohort variable captures the systematic differences introduced by the education delivery format. Additionally, the interactions between cohort and time, as well as team and time, accounted for changes over time and further addressed potential confounding effects related to the transition from in-person to online learning. All analyses were conducted using statistical software R (version 4.2.2).

## 4. Results

There were two research questions proposed for this study.

### Question 1. *How does a two-year curriculum impact IPE perceptions at the all-student level across three student cohorts?*

We investigated changes in IPE perceptions at the all-student level during the two-year curriculum across three student cohorts. The SPICE-R2 mean total score change PY1 and PY2 for Cohorts 2019, 2020, and 2021 (all-student level) are noted in Table 3. Positive changes for all three student cohorts for the total score and each factor (TW, RR, PO). Positive score changes year-over-year were statistically significant according to an observed main effect of time (Table 4). We also observed a main effect of cohort, indicating that subsequent cohorts displayed greater increases in year-over-year score changes (2021 > 2020>2019; Table 4). Lastly, we found significant interactions for the RR and PO factors between time and student cohort (Table 4).

### Question 2. *How does a two-year curriculum impact IPE perceptions at the team level within each student cohort?*

**Fig. 1.**
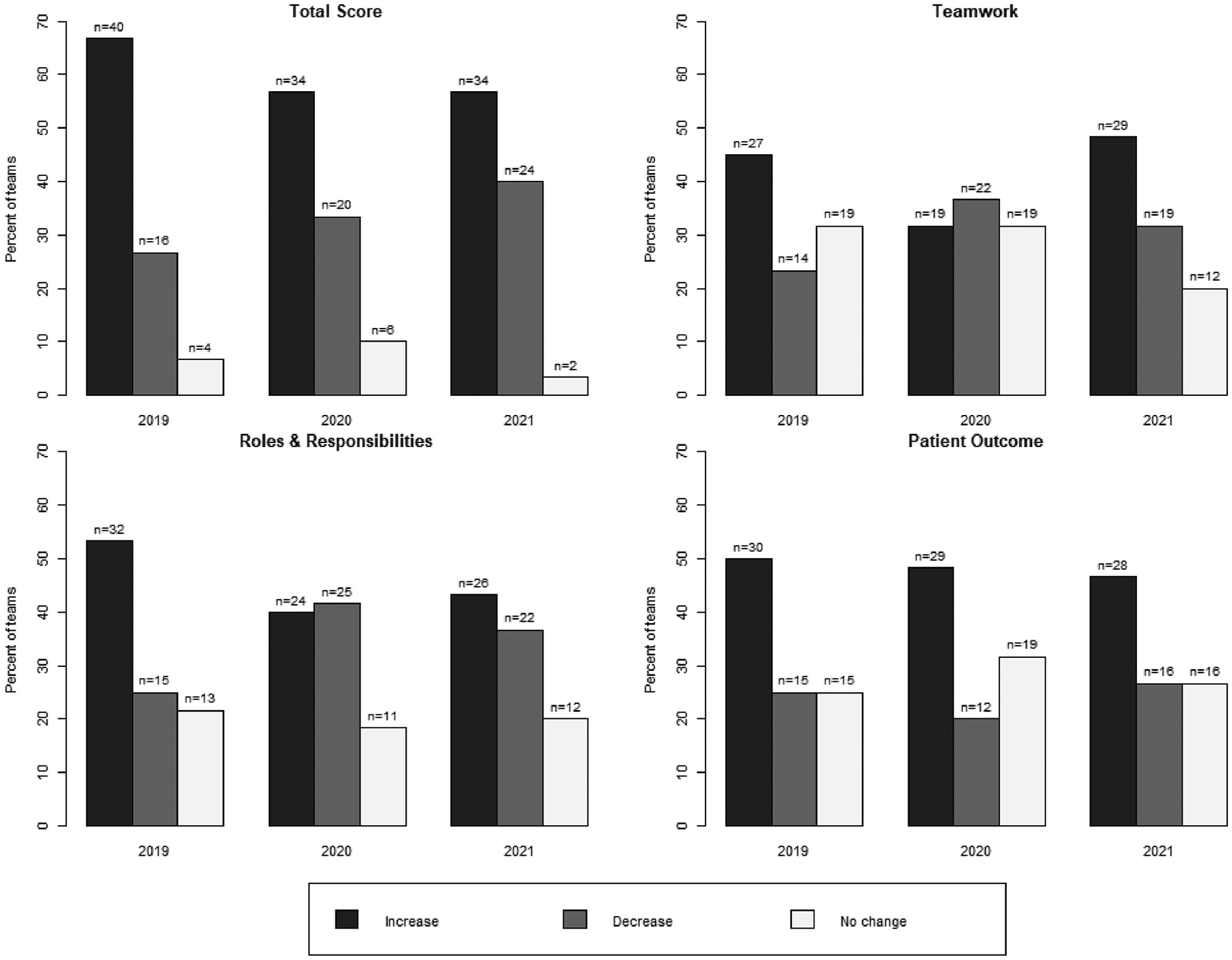
Percent of teams with a change in median score (PY1 to PY2).

**Fig. 2.**
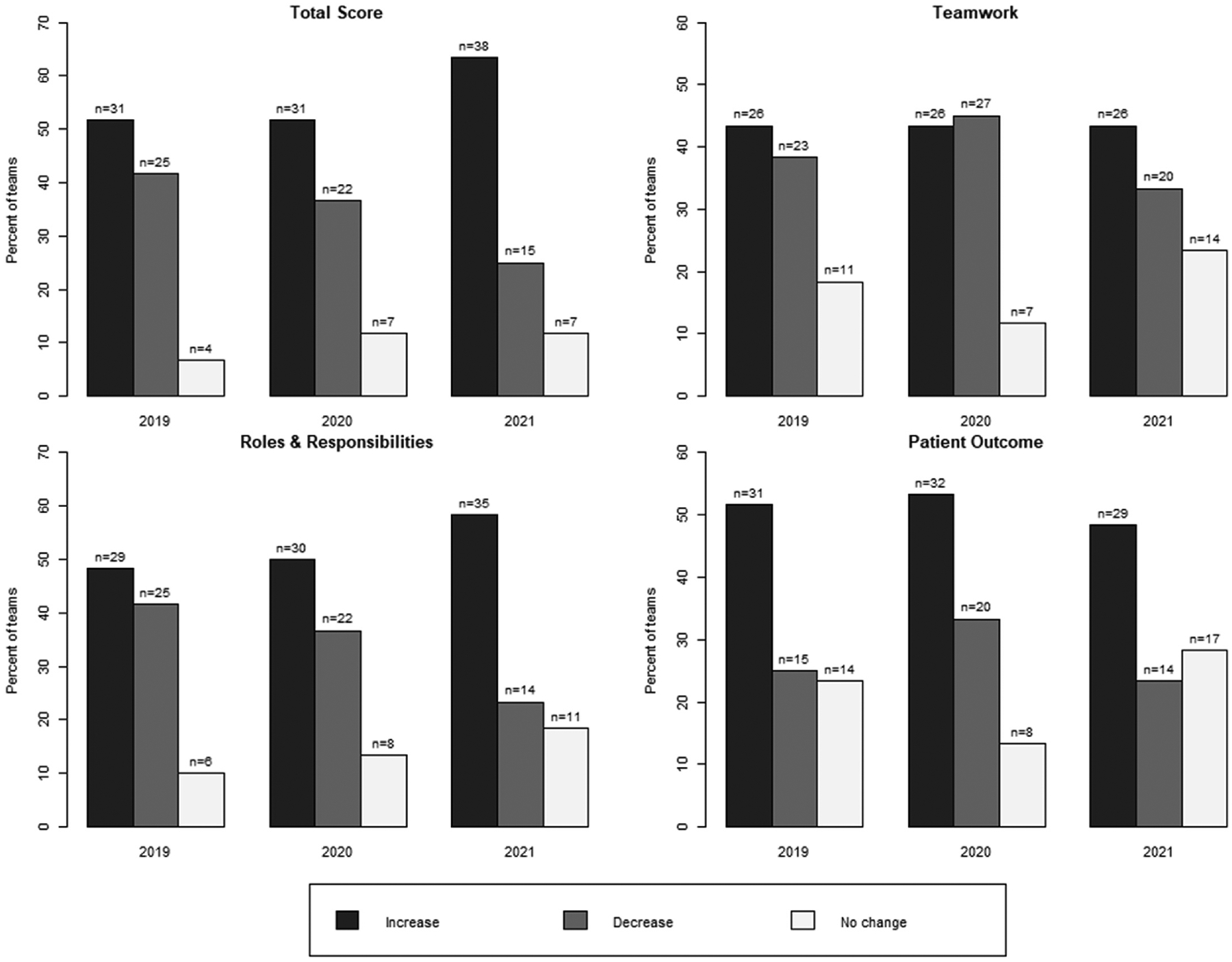
Percent of teams with a change in IQR (PY1 to PY2).

We evaluated the impact of the curriculum on IPE perceptions at the team level across the three student cohorts. Cohort’s team-level analysis are displayed using bar graphs (Figs. 1 and 2) and box plots (Supplement). Figs. 1 and 2 depict the proportion of the 60 teams for each cohort that demonstrated an increase, decrease, or no change in median and IQR (respectively) for total SPICE-R2 score changes. Overall, most teams in all three cohorts experienced a positive change in perceptions (median score change) and increase in variability over time (IQR change over time). Team perception changes varied by cohort by individual construct (TS, TW, PO, RR) measured within the total score. Table 5 indicates an interaction of time and team factors for the total score (TS) and patient outcomes (PO) factor for the student cohort 2020.

## 5. Discussion

This study investigated IPE perception outcomes across three student cohorts at the all-student and team level after engaging in a two-year curriculum. Regardless of the student cohort or time, overall improvements in team perceptions were found to be similar. This finding reinforces that teams consisting of different professions have the opportunity to learn and change perceptions about IPE. Specifically, these teams can improve overall perceptions of IPE.

Findings at the all-student level indicated robust improvements in IPE perceptions. Student IPE perceptions increased after one year and two years of engagement in the curriculum. Additionally, the change in the total SPICE-R2 score significantly increased within each cohort (main effect of time, Table 4). Notably, the change in IPE perceptions increased across the three cohorts from Cohort 2019 to Cohort 2021 (main effect of cohort, Table 4). There were also significant interactions of time and cohort on specific SPICE-R2 factor scores (RR, PO). The classroom and community-based two-year IPE curriculum was designed for many concurrent learners, and the all-student outcomes indicate an overall benefit of the curriculum related to improving IPE perceptions. Furthermore, the consistent increase in score changes over the three sequential student cohorts is promising and potentially reflective of the continuous quality improvement process utilized to refine the TEAM UP^™^ curriculum despite recent environmental challenges.

As an important extension of these preliminary findings, a major focus of the current study was on team-level outcomes. The proportion of teams having an increase in median score change from PY1 to PY2 was approximately 50 % or higher (30 teams or more; Fig. 1) for all three student cohorts. This proportion decreased with student cohorts 2020 and 2021. This study did not investigate factors that could have contributed to this finding. However, the decrease could be related to two years of web-based video learning with Cohort 2020 and the change from web-based video to on-campus learning with Cohort 2021. While not specific to the IPE literature, previous research has noted the social dimension of teamwork is more challenging online.^18–20^ Alternatively, as indicated by year-over-year “baseline” increases in perception changes in PY1, curriculum improvements may have set a limit on further perceptual improvements in Year 2 (Table 3). When viewing the proportion of teams with changes in interquartile range (IQR) scores, IQR ranges increased over time from Cohort 2019 to 2021 (Fig. 2). One would predict that over two years, teams might have more convergent perceptions regarding IPE, regardless of whether these are positive or negative. However, the shifting of education delivery methods to online or hybrid may have influenced this tendency towards divergence over cohorts and time. We explored these possibilities further with additional analyses, and a significant team × time interaction on specific SPICE-R2 score changes (TS, RR) was discovered specifically for the 2020 cohort, indicating a potential influence of environmental factors, like switching to online learning, on team-level outcomes (Table 5).

These findings expand our understanding of the potential for changes in team-level IPE perceptions after engaging in a two-year longitudinal curriculum. Related to formative program evaluation, the findings of this study reveal a need to further investigate beyond team-level perceptions. Evaluating teamwork and communication in preparing learners to be collaborative-practice ready is aligned with the purpose of IPE. However, other considerations exist outside of teamwork and communication for varied perceptions. Two recent IPE studies suggested that intrinsic motivation related to engaging in IPE that focuses on working in an interprofessional team contributes to higher-performing or collaborative teams (Reinder & Krijnen, 2022;^6^). The education delivery modality may also impact outcomes. Many IPE-related research areas that need investigating and preparation for team-based care need further exploration.

This study was conducted following the 2021 Cohort’s graduation from the IPE curriculum. While we did not see statistically significant differences in team-level IPE perception changes (time × cohort), our role as educators is to support the ongoing development of collaborative-practice professionals. It would behoove us to determine how to best support teams that demonstrate a decrease in IPE perceptions, as identified through the SPICE-R2, in a more immediate fashion. This is particularly true when environmental challenges (such as a shift in learning format) arise. Perhaps another measurement time point could be added, a different measurement instrument utilized, or we could identify individuals or teams who indicated a decrease in IPE perceptions post-year 1. Another opportunity to influence perceptions related to IPE is increasing active learning opportunities in the Year 2 curriculum, such as teach-back/return demonstrations, engagement in role-playing or simulated activities, and/or engagement in clinical activities. Lastly, teams could also be partnered with faculty facilitators or professionals who work on interprofessional teams for ongoing assistance and support. These improvements may better build upon the strong increases in SPICE-R2 scores observed PY1 at the all-student level.

Although the findings from this study are from a single-site institution, the results indicate the need to look beyond all-student and program-level outcomes. Some noted strengths of the current study includes using a validated survey instrument (SPICE-R2), the large sample size, and the high response rate and corresponding minimal non-response bias given that the survey was a course requirement. However, some limitations should also be noted. In general, it is difficult to identify confounding individual attitudes nested within teams, as these depend on many mediating effects such as interpersonal processes, trust, and cultural background. For example, student leaders were rotated for each session, which may influence the team members’ buy-in or overall perceptions during each session. Our highly diverse teams were constructed based on school and demographic variables, including graduate and undergraduate learners. Students varied in clinical experiences. Students in programs like the Master’s in Speech-Language Pathology and the Bachelor of Science in Nursing obtained clinical experiences throughout their participation in the IPE curriculum, while other students in programs like the Doctor of Medicine have no mandated clinical experiences. Research has found that pre-clinical students may exhibit increased positive perceptions of IPE compared to those with clinical experience,^21^ which may have influenced individual and team-level attitude changes captured. Individual differences in previous teamwork experiences may have also influenced IPE attitudes. Other factors like drops in overall cohort sizes and changes in grading practices should be noted. Grading shifted to letter grades from a pass/fail determination during the 2021 Cohort. This switch may have influenced individual-and team-level attitudes, especially regarding teamwork and roles/responsibilities factors. The total number of participants in each cohort decreased throughout the study time frame, likely because the School of Public Health program opted out of IPE during the analysis period and due to the potential impacts of the COVID-19 pandemic on enrollment. However, we believe this decrease did not significantly influence the results, as the cohort sizes were relatively similar (675 vs. 633 vs. 601).

In the future, educators should focus on influencing team perceptions with other variables, such as team cohesion, team performance, and team communication (Xyrichis, 2020). Evaluating team-level outcomes like cohesion and communications to improve IPE perceptions and attitudes will be needed to advance interprofessional science. Researchers and educators should look to create consistent and validated pre-post measurement tools capturing team-level constructs. This may allow educators to understand the impact of an institution’s IPE curriculum on students and interprofessional teams.

## 6. Conclusion

In comparison to a previous single cohort study at the same institution,^7^ the results from this multiple cohort study are highly concordant. The IPE literature in team-level research is in its infancy, with limited current evaluative approaches. This exploratory study demonstrated differences in team-level and all-student-level perceptions. The all-student level indicated positive program outcomes for the two-year curriculum. However, team-level outcomes provided a different perspective. If the goal of IPE is to prepare students to effectively work on teams, education-focused researchers should investigate team-level outcomes. We strongly feel that the results of this study provide a good basis for discussion in developing early learners as collaborative-practice ready health professionals.

## Supplementary Material

1

## Figures and Tables

**Table 1:** IPE variable information across three cohorts.

Category	Subcategory	IPE Variable	Description	Cohort One	Cohort Two	Cohort Three
Identifying Situational Factors	General Context of Learning Situation	Program Description	The larger learning context for the program such as being part of an overarching curriculum or as part of an IPE center	This curriculum is required by programs of each of the six colleges and Graduate Studies at LSU Health Science Center New Orleans. This curriculum is housed in the Interprofessional Education and Collaborative Practice Office (IPECP).	This curriculum is required by programs of each of the six colleges and Graduate Studies at LSU Health Science Center New Orleans. This curriculum is housed in the Interprofessional Education and Collaborative Practice Office (IPECP).	This curriculum is required by programs of each of the six colleges and Graduate Studies at LSU Health Science Center New Orleans. This curriculum is housed in the Interprofessional Education and Collaborative Practice Office (IPECP).
		Accreditation Standards	The standards that shaped the program’s development	The Southern Association of Colleges and Schools Commission on Colleges	The Southern Association of Colleges and Schools Commission on Colleges	The Southern Association of Colleges and Schools Commission on Colleges
		Other External Influences	Other external factors that shaped the program’s development	IPEC Competencies, the 7 C’s of Team Science, the meta-model for interprofessional development and the interprofessional socialization framework	IPEC Competencies, the 7 C’s of Team Science, the meta-model for interprofessional development and the interprofessional socialization framework	IPEC Competencies, the 7 C’s of Team Science, the meta-model for interprofessional development and the interprofessional socialization framework
		Institutional Setting	The type(s) of institutions involved such as academic, healthcare, or community-based	LSU Health Science Center New Orleans academic institutions	LSU Health Science Center New Orleans academic institutions	LSU Health Science Center New Orleans academic institutions
	Specific Context of the Teaching/Learning Situation	Geographic Location	Where the program occurred	New Orleans, LA	New Orleans, LA	New Orleans LA
		Dates(s) of Program	When the program occurred from conception to end of the current report	September 2019-April 2020; September 2020-April 2021	September 2020-April 2021; September 2021-April 2022	September 2021-April 2022; September 2022-April 2023
		Durations and Frequencies of Activities	How long each activity was and how often activities happened	6 sessions of content; 1 session of health project presentation/teamwork presentations. Year one sessions are 60 min; Year two sessions are 50 min.	6 sessions of content; 1 session of health project presentation/teamwork presentations. Year one sessions are 60 min; Year two sessions are 50 min.	6 sessions of content; 1 session of health project presentation/teamwork presentations. Year one sessions are 60 min; Year two sessions are 50 min.
		Learner Level	How far along in training the learners were including their level of clinical sophistication and independence	Pre-licensure health students undergraduate and graduate programs. Programs include public health, respiratory therapy, cardiovascular sonography, medical laboratory science, dental hygiene, audiology, physical therapy, medicine, dentistry, physician associate, occupational therapy, speech-language pathology, and undergraduate nursing.	Pre-licensure health students undergraduate and graduate programs. Programs include respiratory therapy, cardiovascular sonography, medical laboratory science, dental hygiene, audiology, physical therapy, medicine, dentistry, physician associate, occupational therapy, speech-language pathology, and undergraduate nursing.	Pre-licensure health students undergraduate and graduate programs. Programs include respiratory therapy, cardiovascular sonography, medical laboratory science, dental hygiene, audiology, physical therapy, medicine, dentistry, physician associate, occupational therapy, speech-language pathology, and undergraduate nursing.
		Approach to Student Engagement	How students were engaged including requirement, grading, and incentives	Requirements of health programs & grades.	Requirements of health programs & grades.	Requirements of health programs & grades.
		Number of Learners per Team	How many learners and of what professional affiliation	Y1 = 791 Y2 = 738;	Y1 = 784 Y2 = 730	Y1 = 709 Y2 = 651
		Other Supports for Learners	How learners were placed on teams including attention to professional diversity	Learners were assigned to teams based on their intended degree and health specialty. The coordinator aimed to create diverse teams with varied health specialties from each of the six colleges.	Learners were assigned to teams based on their intended degree and health specialty. The coordinator aimed to create diverse teams with varied health specialties from each of the six colleges.	Learners were assigned to teams based on their intended degree and health specialty. The coordinator aimed to create diverse teams with varied health specialties from each of the six colleges.
		Modality	What instructional approaches and modalities were used such as simulation, case-based learning, panel discussions, asynchronous work, etc.	Students participated in case-based activity and online asynchronous reading during both Year 1 and Year 2. In Year 1, students participated in the Health Partner Project. Here students connected in-person with community members to listen to an individual’s health care experiences and provide a list of resources to address existing concerns. The final project for Year 2 includes “interprofessional primary or secondary annual wellness visit specific to an age or medical condition.” (^7,15^, pg.4)	Students participated in case-based activity and online asynchronous reading during both Year 1 and Year 2. In Year 1, students participated in the Health Partner Project. Here students connected in-person with community members to listen to an individual’s health care experiences and provide a list of resources to address existing concerns. The final project for Year 2 includes “interprofessional primary or secondary annual wellness visit specific to an age or medical condition.” (^7,15^, pg.4)	Students participated in case-based activity and online asynchronous reading during both Year 1 and Year 2. In Year 1, students participated in the Health Partner Project. Here students connected in-person with community members to listen to an individual’s health care experiences and provide a list of resources to address existing concerns. The final project for Year 2 includes “interprofessional primary or secondary annual wellness visit specific to an age or medical condition.” (^7,15^, pg.4)
			What barriers were present and how were they addressed, even if unsuccessful	COVID-19- Disrupted school attendance and format. TEAM Up courses were moved online for Year 2 of the curriculum.	COVID-19: Impacted school curriculum delivery. Both Year 1 and Year 2 were offered on a virtual platform.	COVID-19 and Returning to in-person environments
		Challenges Encountered and Attempted Solutions	What areas of knowledge were the focus of activities	Immunization, primary and secondary prevention, aphasia, and infection control.	Immunization, primary and secondary prevention, aphasia, and infection control.	Immunization, primary and secondary prevention, aphasia, and infection control.
	Nature of the Subject	Program Topic(s)	How can the community members involved in the activity be described	Community members are involved in the Health Partners Project. Here, members are paired with a team of pre-licensure health students. Community members involved were from around 20 different parishes (counties) in Louisiana, and states like Alaska, Mississippi, and Washington. During their involvement with the project, students listen to these community member’s stories and assist in providing a relevant, up-to-date resource list.	Community members are involved in the Health Partners Project. Here, members are paired with a team of pre-licensure health students. Community members involved were from around 20 different parishes (counties) in Louisiana, and states like Alaska, Mississippi, and Washington. During their involvement with the project, students listen to these community member’s stories and assist in providing a relevant, up-to-date resource list.	Community members are involved in the Health Partners Project. Here, members are paired with a team of pre-licensure health students. Community members involved were from around 20 different parishes (counties) in Louisiana, and states like Alaska, Mississippi, and Washington. During their involvement with the project, students listen to these community member’s stories and assist in providing a relevant, up-to-date resource list.
	Nature of the Subject Characteristics of the Learners	Population Served (if Community-Facing)	Which terminal degrees, licensed professions, or other professional descriptors were represented in the program	Programs include public health, respiratory therapy, cardiovascular sonography, medical laboratory science, dental hygiene, audiology, physical therapy, medicine, dentistry, physician associate, occupational therapy, speech-language pathology, and undergraduate nursing.	Programs include respiratory therapy, cardiovascular sonography, medical laboratory science, dental hygiene, audiology, physical therapy, medicine, dentistry, physician associate, occupational therapy, speech-language pathology, and undergraduate nursing.	Programs include respiratory therapy, cardiovascular sonography, medical laboratory science, dental hygiene, audiology, physical therapy, medicine, dentistry, physician associate, occupational therapy, speech-language pathology, and undergraduate nursing.
		Professional programs	What was the age, race, gender, and other descriptors of the learners	Unknown	Unknown	Unknown
Identifying Learning Goals	Characteristics of the Learners	Learner demographics	How did the learners feel about interprofessional collaboration prior to the activity	Unknown (Conducted a retrospective pre-post test). Students may have had minimal to no experience with interprofessional experience.	Unknown (Conducted a retrospective pre-post test). Students may have had minimal to no experience with interprofessional experience.	Unknown (Conducted a retrospective pre-post test). Students may have had minimal to no experience with interprofessional experience.
		Pre-existing Learner Attitudes	What experience did different learner groups have with IPE or IPC	Minimal to none (not captured)	Minimal to none (not captured)	Minimal to none (not captured)
		Prior exposure to Interprofessional Education and Practice	How many planners and facilitators were involved	7 (six from the faculty council and one from the IPE office)	7 (six from the faculty council and one from the IPE office)	7 (six from the faculty council and one from the IPE office)
		Learning Objectives	What beliefs shaped how the planners constructed activities to meet the learning objectives	IPEC Competencies, the 7 C’s of Team Science, the meta-model for interprofessional development and the interprofessional socialization framework	IPEC Competencies, the 7 C’s of Team Science, the meta-model for interprofessional development and the interprofessional socialization framework	IPEC Competencies, the 7 C’s of Team Science, the meta-model for interprofessional development and the interprofessional socialization framework
		Learning Theory or Framework	How was learner performance measured	SPICE-R2	SPICE-R2	SPICE-R2
Formulating assessment procedures		Outcome Classification	How were learners incentivized to perform at their highest level	Pass/fail	Pass/fail	Grades
Selecting effective learning activities		Planning Approach & Educational Approach	How did the planners develop the program including integration of situational factors, learning goals, theory, and assessment & How did planners and facilitators collaborate to implement instruction, assessment, and evaluation	The curriculum was initially developed during 2015–2017 engaging multiple faculty members from various professions. Since the implementation of the curriculum, the IPECP engaged in a continuous quality improvement approach to improving outcomes. Students can provide feedback via an anonymous survey distributed during both fall and spring semesters for Year 1 and Year 2. An institutional student committee representing all 6 Schools assists the IPECP in prioritizing changes, and a faculty council team representing all 6 Schools collaborate with the IPECP to assess learning and refine the curriculum.	The curriculum was initially developed during 2015–2017 engaging multiple faculty members from various professions. Since the implementation of the curriculum, the IPECP engaged in a continuous quality improvement approach to improving outcomes. Students can provide feedback via an anonymous survey distributed during both fall and spring semesters for Year 1 and Year 2. An institutional student committee representing all 6 Schools assists the IPECP in prioritizing changes, and a faculty council team representing all 6 Schools collaborate with the IPECP to assess learning and refine the curriculum.	The curriculum was initially developed during 2015–2017 engaging multiple faculty members from various professions. Since the implementation of the curriculum, the IPECP engaged in a continuous quality improvement approach to improving outcomes. Students can provide feedback via an anonymous survey distributed during both fall and spring semesters for Year 1 and Year 2. An institutional student committee representing all 6 Schools assists the IPECP in prioritizing changes, and a faculty council team representing all 6 Schools collaborate with the IPECP to assess learning and refine the curriculum.

**Table 2:** Education delivery format by student cohort and academic year semester engagement.

	TEAM UP^™^ Year 1		TEAM UP^™^ Year 2	
	Fall semester	Spring semester	Fall semester	Spring semester
Cohort 2019	2019 On-campus	2020 On-campus	2020 Web-based	2021 Web-based
Cohort 2020	2020 Web-based	2021 Web-based	2021 Web-based	2022 Web-based
Cohort 2021	2021 Web-based	2022 Web-based	2022 On-campus	2023 On-campus

**Table 3:** SPICE-R2 mean score changes for PY1 and PY2, Cohorts 2019, 2020, and 2021 (all students).

	Cohort 2019 Mean (SD) n = 675		Cohort 2020 Mean (SD) n = 633		Cohort 2021 Mean (SD) n = 601	
	PY1	PY2	PY1	PY2	PY1	PY2
Total Score	4.11 (6.35)	5.07 (6.52)	5.28 (7.09)	5.88 (6.89)	6.40 (6.26)	6.87 (7.31)
Teamwork	1.05 (3.03)	1.41 (3.00)	1.52 (3.45)	1.71 (3.14)	1.95 (2.98)	2.18 (3.23)
Roles/Responsibilities	2.15 (2.46)	2.43 (2.46)	2.52 (2.65)	2.58 (2.58)	2.94 (2.47)	2.99 (2.72)
Patient Outcomes	0.91 (1.78)	1.24 (1.91)	1.25 (1.90)	1.59 (1.92)	1.51 (1.80)	1.70 (2.10)

**Table 4:** Two-way repeated measure ANOVA for SPICE-R2 total score and three factors (all-student level).

	Effect	P-value^1^
Total Score	Time	<0.001***
	Cohort	<0.001***
	Time:Cohort	0.058
Teamwork	Time	0.001**
	Cohort	<0.001***
	Time:Cohort	0.089
Roles/Responsibilities	Time	0.004**
	Cohort	<0.001***
	Time:Cohort	0.036*
Patient Outcomes	Time	<0.001***
	Cohort	<0.001***
	Time:Cohort	0.040*

P values obtained from a two-way repeated measure ANOVA (score as response variable; time, cohort and time:cohort as the explanatory variables).

Asterisks indicate p < 0.05*, p < 0.01**, and p < 0.001***.

**Table 5:** Time and team interaction effect on SPICE-R2 score changes.

	Total Score^a^	Teamwork^a^	Roles/Responsibilities^a^	Patient Outcomes^a^
Cohort 2019	0.652	0.747	0.507	0.735
Cohort 2020	0.041*	0.400	0.034*	0.051
Cohort 2021	0.176	0.245	0.317	0.205

a P value corresponding to the effect of the time:team interaction from a two-way repeated measure ANOVA (score as response variable; time, team and time: team as the explanatory variables).

Asterisks indicate p < 0.05*.

## Appendix A. Supplementary data

Supplementary data to this article can be found online at https://doi.org/10.1016/j.xjep.2025.100741.
