# Supplementary material for: Analysis of interprofessional education perceptions at the team level: A study across three student cohorts
Source: J Interprof Educ Pract. Author manuscript; Available in PMC 2026 May 28. (PMC13215709; doi:10.1016/j.xjep.2025.100741)

**Supplement 1**

*Boxplots of the percentage of teams rendering changes in median and interquartile range (IQR)*


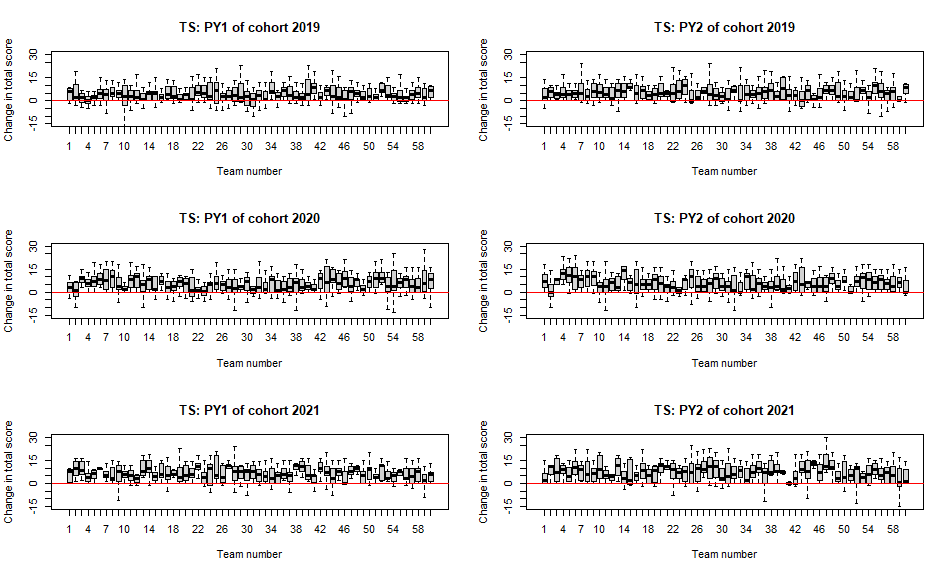


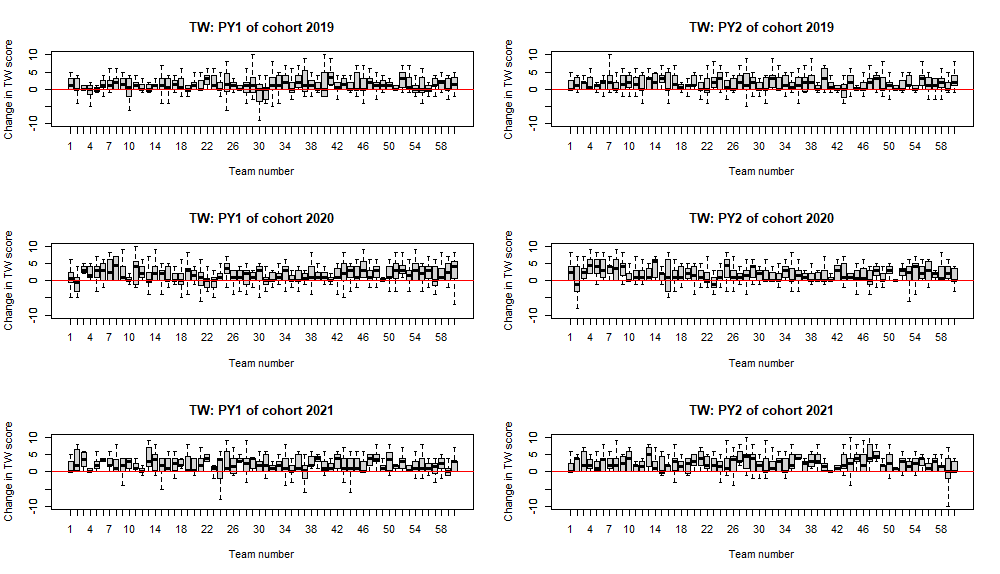


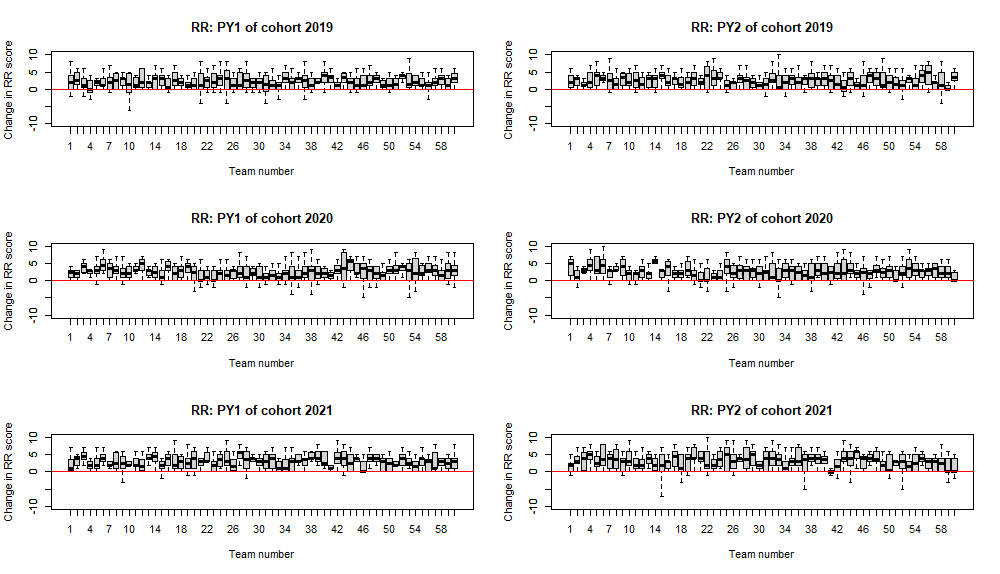


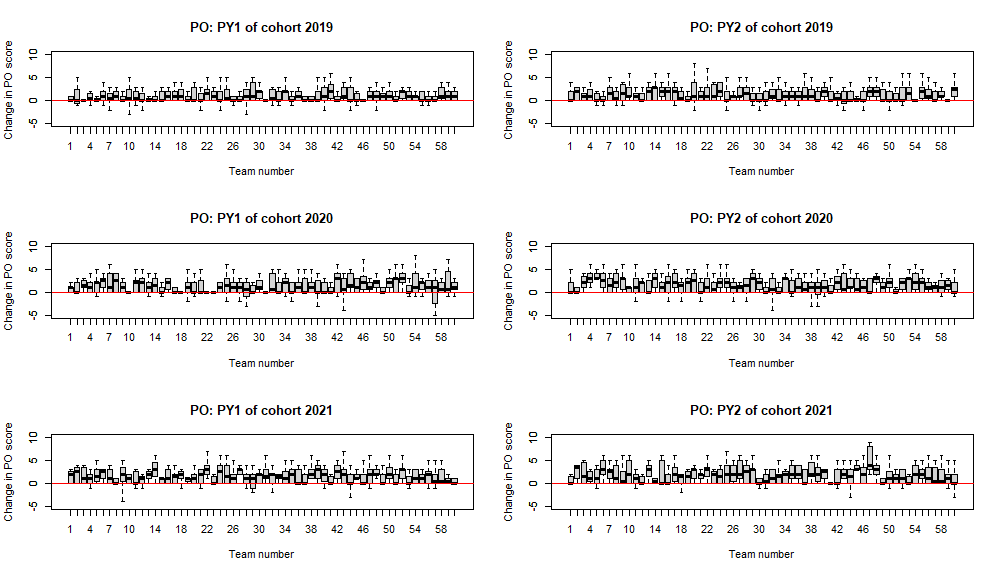

Supplement: 1 [file NIHMS2172273-supplement-1.docx]
